# Supplementary figures and images for: Hyaluronic acid plasma levels during high versus low tidal volume ventilation in a porcine sepsis model
Source: PeerJ. 2022 Jan 5;9:e12649. doi: 10.7717/peerj.12649 (PMC8742546; doi:10.7717/peerj.12649)

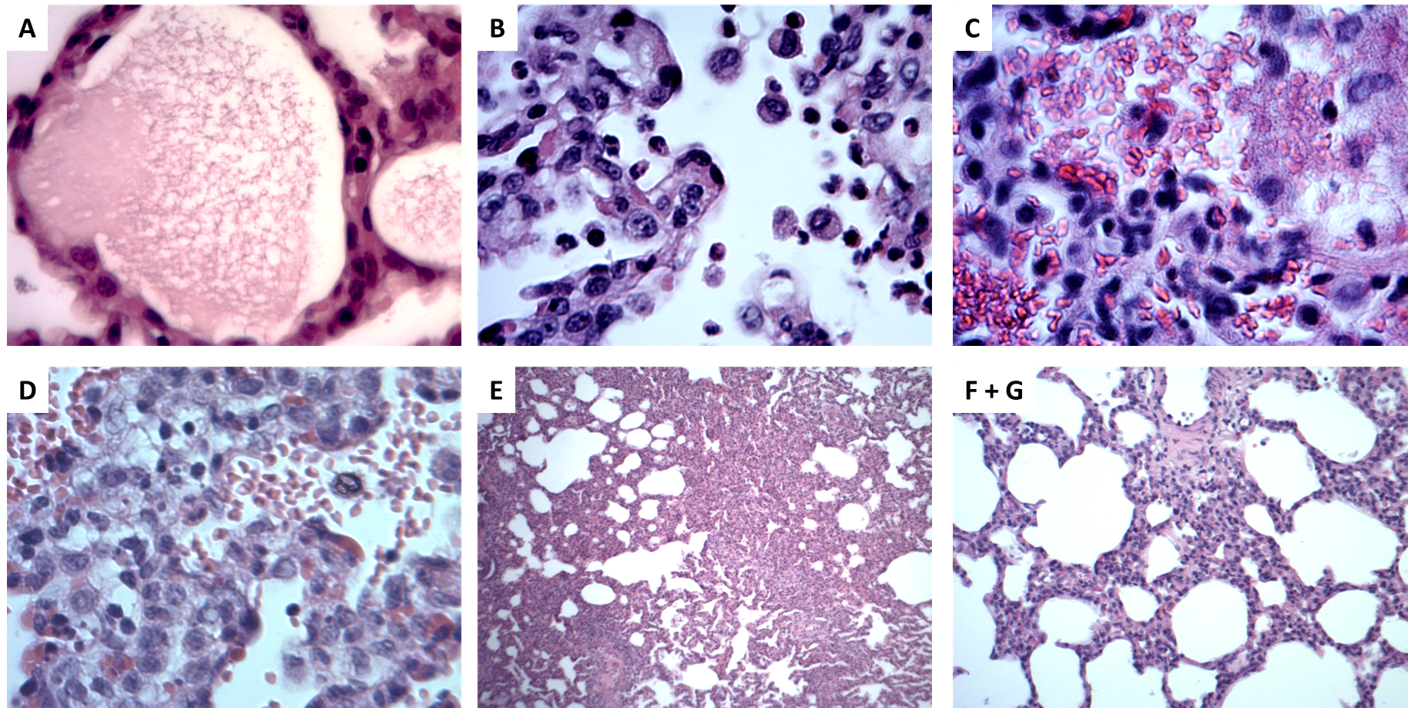

Supplement: Supplemental Information 3 — (extracted and fixed in formalin for parafin sectioning and haematoxylin/eosin staining; image magnification x 10 &20) A: alveolar oedema B: interstitial edema C: hemorrhage D: Inflammatory infiltration E: epithelial destruction F + G: overdistension + microatelectasis [file peerj-10-12649-s003.png]
